# An evaluation of the clinical microsystems approach in general practice quality improvement

**DOI:** 10.1017/S1463423620000158

**Published:** 2020-06-23

**Authors:** Vanessa Abrahamson, Sabrena Jaswal, Patricia M. Wilson

**Affiliations:** 1 Research Associate, Centre for Health Services Studies, University of Kent, Canterbury CT2 7NF, UK; 2 Professor of Primary and Community Care, Centre for Health Services Studies, University of Kent, Canterbury CT2 7NF, UK

**Keywords:** general practice, primary healthcare, quality improvement, qualitative research, implementation science

## Abstract

**Background:**

Changes to the general practice (GP) contract in England (April 2019) introduced a new quality improvement (QI) domain. The clinical microsystems programme is an approach to QI with limited evidence in primary care.

**Aim:**

To explore experiences of GP staff participating in a clinical microsystems programme.

**Design and setting:**

GPs within one clinical commissioning group (CCG) in South East England. Normalisation process theory informed qualitative approach.

**Method:**

Review of all CCG clinical microsystems projects using pre-existing data. The Diffusion of Innovation Cycle was used to inform the sampling frame and GPs were invited to participate in interviews or focus groups. Ten practices participated; 11 coaches and 16 staff were interviewed.

**Results:**

The majority of projects were *process*-*driven* activities related to administrative systems. Projects directly related to *health outputs* were fewer and related to externally imposed targets. Four key elements facilitated practices to engage: feeling in control; receiving enhanced service payment; having a senior staff member championing the approach; and good practice–coach relationship. There appeared to be three key benefits in addition to project-specific ones: improved working relationships between CCG and practice; more cohesive practice team; and time to reflect.

**Conclusion:**

Small projects with clear parameters were more successful than larger ones or those spanning organisations. However, there was little evidence suggesting the key benefits were unique attributes of the microsystems approach and sustainability was problematic. Future research should focus on cross-organisational approaches to QI and identify what, if any, added value the approach provides.

## Introduction

The UK’s National Health Service (NHS) England recently agreed a new five-year framework for general practice (GP) contract reform (NHS England, 2019a) to implement The NHS Long-Term Plan, an ambitious strategy to improve the quality of patient care and health outcomes (NHS England, 2019b). The Quality and Outcomes Framework (QOF) is a voluntary scheme and aims to support GPs to deliver good quality care; the indicators, or measures, are agreed as part of the GP contract negotiations every year and against which performance is measured (NHS England, 2019a). Changes to the contract in April 2019 introduced a new quality improvement (QI) domain with specific indicators for prescribing and end-of-life care; GPs are expected to recognise areas of care which require improvement and ‘address this through a QI plan and sharing of learning across their network’ (NHS England, 2019a, p. 96).

The clinical microsystems (CMS) approach to QI is underpinned by systems theory and complexity science (Nelson *et al*., 2008). A clinical microsystem is defined as a ‘small group of people who work together on a regular basis to provide care to discrete subpopulation of patients’ (Nelson *et al*., 2002, p. 474). It is a functioning unit which produces services that can be measured as performance outcomes (Foster *et al*., 2007). GPs are distinct clinical practice units with a designated purpose and function, fitting this definition well (Nemeth *et al*., 2008). The CMS process involves identifying a problem and developing a systematic approach to address it using a ‘toolkit’ of activities and a workbook to lead the team through a process (Godfrey *et al*., 2010). Table 1 outlines key steps and terminology.

**Table 1.** Five steps of the clinical microsystems approach

Step	Key activities
Step 1: Organise a ‘lead team’	Assemble a ‘lead team’ to represent all disciplines and roles in the practice (professional and clerical), and patients. Employ effective meeting skills, including assigning individual roles/tasks. Meets weekly to maintain focus, plan and oversee improvement work
Step 2: Do the 5Ps assessment	Complete the ‘5Ps’ assessment of Purpose, Patients, Professionals, Processes and Patterns using practice data and the microsystems workbook with templates. For example, fishbone diagram (cause and effect analysis) and data wall (metrics related to problem being addressed). Aims to create an overview of the system under review and identify improvement opportunities
Step 3: Make a diagnosis	Review the data (including strengths and weaknesses of the system) and select an issue to address. Create an overall theme, or global aim statement to maintain motivation and focus
Step 4: Treat the microsystem	This begins with making a specific aim statement using numerical goals, specific dates and specific measures. Uses Plan-Do-Study-Act (PDSA) as the model for improvement. Address sustainability issues using Standardize-Do-Study-Act (SDSA). Daily huddle whereby the team reviews the coming day/week to plan actions based on patient need and available resources, and contingency planning
Step 5: Follow-up	Monitor the new patterns of results and select new themes for improvement. Embed new habits into daily work using daily huddles, weekly lead team meetings, monthly all team meetings, data walls and storyboards

Source from Godfrey *et al.* 2010.

CMS are usually part of a larger organisation within the mesosystem. Each person’s healthcare is likely to involve a number of CMS that should fit together to provide seamless care (Nelson *et al*., 2008). However, social policy has predominantly focused on the organisational level and individual provider level, thus missing the potential contributions of microsystems to patient outcomes (Mohr and Batalden, 2002).

Most health and care services in the UK are commissioned by groups of GPs known as clinical commissioning groups (CCGs). From June 2019, all GPs were also required to align themselves to a larger primary care network (PCN), covering 30 000–50 000 patients. The networks are intended to provide the structure and funding for services to be developed locally (NHS England, 2019c). This study was commissioned by a CCG in South East England that introduced CMS within its member practices and supported a funding stream to enable this. Given the emphasis on delivering significant levels of care outside hospital (NHS England, 2019c), the CCG wanted to enable GPs to manage demand, strengthen their workforce and support struggling practices. Prior to carrying out the evaluation, we reviewed the literature on CMS; there were few studies based in GP or in the UK so we widened our review to all CMS literature (Supplementary information 1). Many issues raised in the wider literature, such as difficulties working across microsystems, were relevant to our setting and helped inform the evaluation.

Most of the CMS literature stems from a series of papers in different areas of healthcare in North America (Nelson *et al*., 2002; Nelson *et al*., 2003; Godfrey *et al*., 2003; Wasson *et al*., 2003; Batalden *et al*., 2003; Mohr et al., 2003; Kosnik and Espinosa, 2003; Huber *et al*., 2003; Batalden *et al*., 2003; Nelson *et al*., 2008; Wasson *et al*., 2008; Godfrey *et al*., 2008; McKinley *et al*., 2008). More recent UK and global examples are largely within the hospital environment (Batalden *et al*., 2003; Likosky, 2014) and it is difficult to ascertain what improvements are directly attributable to the approach (Godfrey *et al*., 2008).

Although there are some examples of CMS informed QI in the UK (Gill and Gray, 2006; Williams *et al*., 2009; Risi *et al*., 2015; Gerrish *et al*., 2018), only Risi *et al*. (2015) is specific to GPs. Although it recruited just five practices, had mixed findings and details of the method were limited, the study reported positive outcomes in terms of patient and staff satisfaction. In particular, working in small teams enabled ‘the best aspects of small practice working’ under the ‘umbrella’ of the wider organisation (Risi *et al*., 2015, p. 536).

Williams *et al*. (2009; 2007) highlighted similar benefits of CMS in six different NHS settings. This comprehensive realist evaluation focused on context and process, as would be expected, but the findings were limited by a lack of data on patient outcomes. However, CMS implementation was ‘unanimously seen as having led to improved communication’ and teams ‘developed greater cohesiveness, mutual support and team building’ (Williams *et al*., 2009, pp. 124–5). A key implementation issue appeared to be how the approach was initially presented to clinicians so that it ‘captures their interest and pushes them to engage, and then sustains credibility’, alluding to human agency. However, there is limited research into the interplay between individuals and the organisation within which they work. The Consolidated Framework for Implementation Research (CFIR) was developed with this in mind (Damschroder *et al*., 2009). One of the five domains, individual characteristics, addresses ‘how individuals perceive the organisation and their relationship and degree of commitment with that organisation’; factors that could affect implementation include different priorities, attitudes to learning and the implementation climate (Damschroder *et al*., 2009), all pertinent to our evaluation.

Two studies in primary care in the USA, albeit a different context to the UK, were both process-driven: improvement in waiting room times using pre- and post-test patient surveys, which demonstrated increased patient satisfaction (Michael *et al*., 2013) and implementing clinical guidelines for prevention of cardiovascular disease using a common electronic record (Nemeth *et al*., 2008). The CMS approach provided a mechanism to develop deeper understanding of the process of change; practices were most effective when they had clear vision and goals, team involvement, and opportunity to feedback and modify the goals (Nemeth *et al*., 2008). These aspects were absent in an analysis of the hospital to community interface in the Netherlands (Gobel *et al*., 2012) and the approach highlighted communication barriers between inpatient teams and GPs which negatively affected the quality of patient transitions. Although individual clinicians did their best, the results were inadequate because they worked in isolation, both sides, or CMS, failing to communicate effectively across the mesosystem (Gobel *et al*., 2012).

Finally, two evaluations of CMS in Australia examined success characteristics of high-performing GPs (Dunham *et al*., 2018) and the adoption of a diabetes care delivery programme in primary care (Janamian *et al*., 2014). Both highlighted the importance of leadership at the micro-level alongside support at the macro-level. Similarly, staff focus with education and training was essential, including inter-professional (or team-based) learning. Janamian *et al*., (2014) highlighted that the approach was effective in promoting innovation in primary care because ‘it offers a way to integrate structure, process and outcomes of care’. While staff liked the use of ‘real data’, provided by the toolkit (5Ps exercises), it was the link to patient care that triggered adoption.

Potential benefits, barriers and facilitators are summarised in Table 2.

**Table 2.** Benefits, facilitators and barriers to the CMS approach in general practice

	UK (Williams *et al*., 2009; Risi *et al*., 2015)	USA (Nemeth *et al*., 2008; Michael *et al*., 2013)	Australia (Dunham *et al*., 2018; Janamian et al., 2014)	The Netherlands (Gobel *et al*., 2012)
Benefits of CMS approach	Improved staff morale, empowerment, commitment and clarity of purpose Shift in culture towards a more active approach to individual and collective improvement Greater awareness of the practice’s function and individual roles to deliver it Improved communication within the team Reduced GP isolation and better emotional support Identifying and nurturing strengths, of both teams and individuals Greater capacity to manage externally imposed change			
Facilitators	Inclusive leadership and Interdependence of the team Buy-in from all staff Maintaining a staff focus with investment in staff training and support Identification of champions of change Celebration of positive achievements The use of ‘real data’ to demonstrate improved outcomes External input to support change management Infrastructure to support teams/information technology			
Barriers	Lack of staff buy-in, scepticism or dissent High staff turnover Communication difficulties within and across teams An inability to grasp the interdependencies of the system			

The CCG that this study focuses on introduced CMS in 2013 and trained nearly 20 CCG managers as coaches. Twenty-two projects were initiated in GP, with a further four in acute hospitals and another bridging four community hospitals. CCG managers were matched with GPs who expressed interest in the initiative; practices received a reimbursement package for their first project. The CCG collected detailed process and outcome data, including the challenges and QI indicators specific to each project’s aims and objectives. This subsequent evaluation focuses on the perceptions of GP staff and fills a gap in the literature specific to GPs in England, in the context of current policy requirements (NHS England, 2019a; 2019c). It aimed to ascertain:

1. What are stakeholder perceptions of the clinical microsystems methodology?
2. What are the facilitators and barriers to embedding the clinical microsystems methodology into GP?
3. How did the reimbursement package trigger adoption of the programme and is it required for sustained adoption?

## Method

### Design, setting and participants

A review of all CMS projects carried out within the CCG was undertaken using the existing data collected by the CCG. We used Rogers (2010) Diffusion of Innovation Cycle to inform our sampling strategy. The model is used to explain how, over time, an idea gains traction and is adopted by a specific population. Rogers (2010) proposed five categories: ‘innovators’ who are the first to try new approaches and are willing to take risks (akin to CCG leads who championed CMS); ‘early adopters’ who enjoy leadership roles and embrace opportunity for change; ‘early majority’ who adopt change sooner than average; ‘late majority’ who are sceptical of change but will adopt a new approach provided there is evidence; and ‘laggards’, the most sceptical and resistant to change. We divided our sample into three groups: those who had engaged with the approach at the first opportunity (‘early adopters’ and ‘early majority’, combined in the results section); those who had recently completed their first project or were undertaking a project during the evaluation period (‘late majority’); and practices that had withdrawn or declined to participate (‘laggards’). We aimed to sample three practices from each of these categories and to interview two to three people within each practice including a GP and practice manager.

### Research team and data collection

All interviews were carried out by two researchers between June and September 2018. The CCG invited potential practices to participate and asked them to contact the researchers directly. For pragmatic reasons, participants were given the choice of interviews (in person or by telephone) or focus groups (carried out at the practice). Interviews lasted approximately 30 min and focus groups an hour. In-depth interviews were also carried out with coaches delivering the intervention. The project was granted ethical approval by University of Kent. Informed consent was taken prior to each interview.

The topic guide (Table 3) was informed by normalisation process theory (NPT) (May *et al*., 2009). NPT provides a robust methodological approach to understanding how well a complex intervention has been embedded in everyday practice and is used extensively in health service evaluation. We divided the topic guide into the four main categories of NPT: sense-making, or understanding the purpose of CMS; cognitive participation, or buy-in; action, or carrying out the required tasks; and reflexive monitoring/evaluation, both individually and collectively. The guide was designed to facilitate in-depth contextual evaluation exploring the extent, enablers and barriers to the implementation of the approach, indications of culture change and the impact on GP. It was modified for practices that withdrew or declined to participate (Supplementary information 2). Similarly, the topic guide for coaches reflects those for GPs and is available as Supplementary information 3.

**Table 3.** Topic guide for GPs that participated

NPT area	Question and prompts
	To start off, could you tell me a little about yourself? 1. Your role and how long you have been at the practice 2. Your knowledge and/or experience of clinical microsystems prior to the programme 3. Your involvement in the clinical microsystems programme 4. Tell me about the project(s) you were involved with
Sense making/coherence	What were your expectations for the clinical microsystems programme? 5. What were you hoping the programme would bring to the practice? 6. Why did you feel there was a need for the programme? 7. Did you feel well prepared about what it would involve for the practice?
Participation/engagement	Was there ‘buy-in’ to the programme? 8. Were all staff in the practice happy to be involved in the programme? Did this change over time? 9. Did someone in the practice have to champion it? 10. How important is the enhanced service payment for buy-in? Does it need to be continued to sustain buy-in?
Action/doing	What did the programme actually require the practice to do? 11. How much time did this take? What activities did you have to undertake? 12. How helpful were specific tasks related to mapping the 5Ps? 13. Can you tell me about the coach? 14. How would you describe their approach? 15. How important was the coach? 16. What was their relationship with the practice? 17. How often did you see them? How did they maintain contact? 18. Could they have done things differently?
Appraisal	Finally, what value you think the programme provided? 19. Were there any key ingredients that you think made the *project* successful? OR Were there any key ingredients lacking from the *project* that hindered its success? 20. What aspects of the clinical microsystems *approach overall* do you think are essential? 21. How would you describe the changes the programme bought for the practice? 22. Who has benefited most from the programme and how? 23. How do you think the programme equips your practice for future challenges? 24. Would you recommend the programme to others? Can you elaborate? 25. How could the programme be improved?

### Analysis

All interviews and focus groups were digitally recorded, transcribed and anonymised. NPT was used to structure a framework to code and analyse data in Nvivo (version 11). Coding was carried out by two researchers (VA and SJ). The coding frame and emerging themes were discussed and developed over several meetings with the chief investigator (PW). Comparative case-study analysis was used to identify and explain patterns across the different projects. Although data saturation was not anticipated, it was reached in terms of theoretical saturation, or the point in data collection when no additional insights emerge and conceptual categories are considered ‘saturated’ (Corbin and Strauss, 2015).

## Results

Eleven coaches were interviewed and included those with managerial, service development and QI roles. Most had coached in different clinical areas to that of their commissioning role. Coaches had varying levels of experience from having carried out just one project with supervision to having carried out several and/or mentored other coaches. To protect confidentiality, coaches will be referred to as C1-11. Ten GPs and 16 staff (P1-16) participated. Table 4 summarises the role of staff, the approach to change (Rogers, 2010) and whether they participated in an interview, dyad or focus group; coaches are similarly categorised, according to the type of practice they coached. As anticipated, it was not possible to interview more than one person from practices that withdrew or declined to participate. To protect confidentiality, minimal information is provided about projects and participants.

**Table 4.** Practices and coaches that participated

Practice:	Participant’s role	‘Early adopter’	‘Late majority’	Withdrew or declined
A^a^	P1: Practice manager P2: Care coordinator/administrator	✓		
B	P3: GP			✓
C	P4: GP/wider remit (education)			✓
D^a^	P5: Patient services manager P6: Assistant practice manager		✓	
E	P7: GP	✓		
F^a^	P8: GP P9: Secretarial, prescription clerk and CMS coordinator		✓	
G	P10: GP	✓		
H	P11: Patient services manager		✓	
I	P12: Practice manager	✓		
J^b^	P13: Practice nurse P14: Administrative P15: Practice nurse P16: GP		✓	
Coaches:	Experience of coaching by GP category and involvement in mentoring new coaches			
C1	Experienced coach and mentor	✓	✓	
C2	Trained more recently; co-coached with an experienced mentor			✓
C3	Experienced coach; one project was across microsystems	✓	✓	✓
C4	Experienced coach and mentor	✓	✓	
C5	Trained more recently		✓	
C6	Experienced coach and mentor	✓	✓	
C7	Experienced coach	✓	✓	
C8	Trained more recently		✓	
C9	Experienced coach	✓		✓
C10	Experienced coach and mentor	✓	✓	

a Dyad interview.

b Focus group.

**Figure 1.**
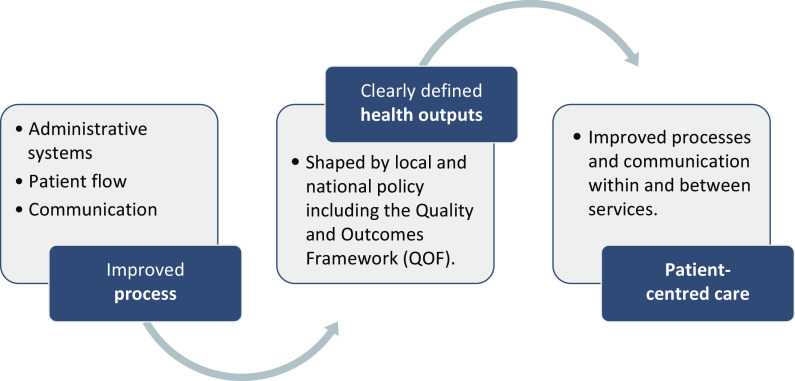
Typology of clinical microsystem projects.

The majority of projects were *process*-*driven* activities related to administrative systems, patient flow and communication. These projects often benefited frontline staff dealing with complicated and overlapping processes. Projects directly related to *health outputs* were much fewer and usually related to QOF targets, such as annual checks for diabetic patients. Projects that aimed to focus on *patient-centred care*, such as a one-stop clinic for those with long-term conditions, could also be categorised by process or outcomes, for example, streamlining the process for identifying and inviting people with long-term conditions to a yearly review. Patient and public involvement (PPI) was limited, which respondents attributed to the process-driven nature of most projects and difficulty engaging patients. The overlap between categories is not surprising given that improved processes are likely to benefit the patient experience, as Figure 1 demonstrates.

### Stakeholder expectations and aspirations

This section explores the expectations of GP staff, how they conceptualised the microsystems approach and its relevance to QI. Most GPs signed up to the approach after learning about it at CCG events or from informal conversations with commissioners. Early adopters were particularly enthusiastic and were keen to include the whole team:

*I think it’s a brilliant idea and it does definitely seem to promote change relatively quickly… because we tend to be very doctor-led within practices* (P7, GP, early adopter)

However, late adopters and practices that withdrew were sceptical. For example, Respondent 4, a GP, regarded CMS as an evidence-based approach that would have benefited the practice but the project floundered due to lack of support from colleagues. The most negative view was that the approach had been imposed on the practice and was unsuited to their context:

*The practice is well aware of where the difficulties are and we want a local solution… we don’t want an external person coming in to tell us how to run the system… I think the theory was taken from the States in a hospital setting and I don’t think that relates to general practice so, I’m not sure who generated the idea, but I think it was definitely generated by management rather than by general practice* (P3, GP, withdrew)

Practices that engaged (early adopters and late majority) were looking for a way to address long-standing problems. The microsystems programme was regarded as a valuable opportunity to address either a specific issue, mostly process-driven, such as establishing an effective method of managing patients’ incoming test results within a specific time frame or to develop a new patient-focused initiative, such as a weight loss clinic individualised to the needs of a specific group. Early adopters spotted a wider remit for microsystems that incorporated improving team cohesion and developing their skill set. Conversely, practices that withdrew regarded the approach as one more externally imposed demand on their time. They were focused on tangible outcomes such as efficiency savings and when these were not forthcoming, the approach was discontinued.

Buy-in within practices varied. Early adopters all sustained a positive outlook while those that withdrew started with, and maintained, negative views. The late majority expressed mixed opinions and this sometimes resulted in discord; however, some respondents changed their mind:

*I had very low expectations… It sounded very American… but once we got it started it was a really helpful exercise* (P13, practice nurse, late majority)

Where there was discord, the approach was regarded as an opportunity to redress internal politics and rebuild fragmented relationships:

*The last practice manager had gone, there’d been a period with… no real leadership and the staff team was very fragmented… this was a chance to come together and work on something as a group* (P1, practice manager (PM), early adopter)

Coaches regarded the microsystems approach as a way of supporting GPs to build an ethos of continuous QI. They wanted to build strong relationships with practices; demonstrate that they understood the pressures of practice life; and help staff to develop relevant skills and a culture of reflection that facilitated them to take control of their own improvement:

*This was exactly why we wanted Microsystems in there, because we wanted the practice to be left with tools that would allow them to be more imaginative, transformational, and then include quality in their own environment* (C10)

Most participants liked the toolkit that involved a series of tasks because they could see progression and this contributed to maintaining a safe forum where views could be expressed. Tasks that participants commented were helpful included the ‘5Ps’, the fishbone (visual representation of the issues) and the Plan-Do-Study-Act (PDSA) cycle (see Table 1), although the more sceptical questioned the benefits of being ‘sold this product’ (P15, practice nurse, late majority):

*I do wonder whether the Microsystems approach has a level of complexity that is, perhaps, unnecessary* (P4, GP, withdrew)

Common criticisms, mostly from the late majority and those that withdrew, were that there was too much theory, the terminology was off-putting, the process was unnecessarily rigid and took too long. These criticisms were countered by those who liked the structure, found the workbooks helpful and perceived the toolkit as ensuring a thorough approach. A more nuanced view was that the process was less important than an overall commitment to change:

*Having a structure was helpful… whether it precisely had to be that structure I’m not sure. I think the fact that there was a coach and there was a commitment to progressing was probably more important…* (P10, GP, early adopter)

### Aspects of the approach that facilitated participation

There appeared to be four key elements that facilitated engagement with the contrary experience perceived as a barrier, mostly by the late majority and practices that withdrew. First, practice staff needed to feel that they were in control of the agenda. The coach’s role was to *facilitate* identification of a project but not to decide what that project should be or how to address it. Often, the project related to a long-standing problem which staff wanted to remediate. Coaches understood this and wanted to support practices to set their own agenda:

*It’s about the practices deciding what the challenges are and coming up with a solution and… empowering them and them feeling part of the decision-making process* (C7)

However, participation was limited if staff felt ‘coerced’ into accepting the programme or had prior unsuccessful attempts at solving the problem. The programme needed the support of at least one senior partner to become established, as was the case with all early adopters, but was also regarded as a bottom-up approach that would appeal to junior staff:

*There was some pessimism amongst some of the partners and management staff… it seemed like a good opportunity to put in place a process that was slightly at arm’s length from a top-down management and hopefully get the staff on board* (P10, GP, early adopter)

Secondly, the enhanced service payment appeared essential for practices to engage in the initial project. The payment allowed staff to be released for meetings and allayed anxiety that colleagues would perceive this as wasted time. Not all practices used the money for a locum, either because they could not find one or because they used the money in other ways, but it was an incentive, albeit not sufficiently so for practices that later withdrew:

*[It was] reasonably generous and proportionate for the time taken, but it wouldn’t actually give us workforce, because actually locums are very hard to come by… it was… an incentive* (P4, GP, withdrew)

In addition, the payment helped lend the project credibility, improved buy-in from those who had reservations and facilitated completion:

*It gave it kudos to the partners because they’re obviously the ones who have got to make the decision whether we put the time into it… I don’t think they would have been as welcoming to it had they not received that [reimbursement]* (P11, patient services manager, late majority)

Thirdly, all successful projects needed at least one staff member who ‘championed’ the approach and supported the coach:

*You do need champions… you need an enthusiast who will fly your flag and if there’s a bit of negativity… if you’ve got a positive role model that really helps to get everyone on-board* (C4)

The champion did not have to be a senior clinician and some thought it preferable not to be the GP, given that GPs had insufficient time and could be difficult to challenge. Successful projects included all staff and this went hand in hand with the role of champion and challenging hierarchical boundaries. Where the team had experienced internal conflict, this made it harder for the champion to maintain momentum. Either practices that withdrew did not have a champion (P3) or colleagues (P4) overruled the champion. Communication between the microsystem and the wider practice team was also important to maintain buy-in from those not directly involved. When a project crossed organisation boundaries, a strong champion and coach were needed to draw the two teams together and find mutually beneficial ways of addressing problems. When buy-in from one team was limited, this resulted in less successful outcomes and/or lack of sustainability.

Fourthly, a good working relationship with the coach, built on trust and mutual understanding, was essential. When the coach had prior experience that was deemed relevant, this helped cement the relationship and staff appreciated the coach’s skills:

*She was very good… really helpful, really supportive, she brought ideas from what other practices had done… she was really informative and gave us good encouragement and kept us on track* (P11, patient services manager, late majority)

Practice staff were ambivalent about the level of expertise needed to coach effectively. A lack of relevant experience was regarded as a limitation but not necessarily detrimental to the project if the coach was an effective facilitator. There were mixed views about the merits of potentially training practice staff to become ‘internal’ coaches. Many staff favoured an external (‘objective’) coach who was able to challenge entrenched hierarchies and mediate disagreements:

*I think external is always good because they have no preconceived ideas of hierarchy… she could just say what she thought… if it was a receptionist trying to say something to a senior partner that would be tricky* (P7, GP, early adopter)

Additionally, an external coach was preferable because they brought benefits as an outsider, including the PPI perspective:

*I think first of all having somebody who’s outside of the practice is vital to it because they keep you on track and they’re fresh eyes in the whole process, so they ask questions that possibly a patient would ask whereas we just assume that’s what it should be* (R11, patient services manager, late majority)

However, both practices that withdrew (R3-4) felt that ‘we could of done it quicker ourselves’ (R4, GP) and achieved similar outcomes.

### Outcomes, sustainability and embedding into practice

Project-specific outcomes (such as the number of diabetic annual reviews per month) had been evaluated by the CCG and are not the focus here. There appeared to be three main benefits in addition to project-specific ones, commented on by early adopters and late majority. First, as already alluded to, successful projects helped foster positive working relationships between the CCG and the practice and this supported ongoing and mutually beneficial communication:

*Having a coach from the CCG coming in built a relationship between me and the CCG… [the practice] has continued to benefit over and over again because of that relationship* (P1, PM, early adopter)

Secondly, the approach appeared to benefit relationships within the practice, challenged hierarchies and allowed frontline staff to feel valued and listened to:

*I learnt… about involving a wider team, about getting buy-in from the people on the ground who will need to be implementing any solution, about perhaps being less hierarchical about things* (P10, GP, early adopter)

Thirdly, the programme allowed staff time out from everyday pressures to reflect on processes that they had long adhered to, as this practice manager identified:

*It gives you the chance to sit back, look, think about it, assess it, how can we do it better* (P1, PM, early adopter)

However, successful projects required considerable time and commitment, frequently more than was anticipated, and often required staff to work outside work hours. Sustainability rested on time, motivation and ‘having the right personnel in place to make it work’ (P8, GP, late majority). Where staff changed or had too many competing demands, things slipped, especially when it involved an intervention, such as diabetes annual checks:

*The outcomes would have been sustainable if the team hadn’t changed… our model was dependent on the skill mix that we had at that time and then the doctor left so we got a new doctor who wasn’t so happy with the way that we’d set things up and then the nursing staff changed as well* (P7, GP, early adopter)

Similarly, another practice had started a weight management initiative which was working well, but when external funding was cut the project ceased which left staff feeling the work had been wasted:

*It was all very enthusiastic but… there was no funding… it all came out to a blank… if you can’t offer the service at the end, what’s the point, that’s the main frustration for us* (R13, practice nurse, late majority)

Sustainability was particularly difficult when a project involved two organisations and staff changes upset the relationship between organisations and was detrimental to outcomes. Across all categories, in the context of competing demands, staff tended to revert to previous familiar methods:

*Inevitably people’s enthusiasm dies… we were changing things that had been done in the practice the same way for years and so people then just slip back to doing what they’ve been doing before* (P7, GP, early adopter)

Opinions were mixed about embedding the approach into practice with even early adopters struggling to achieve this. The most negative perception (expressed by some late majority, and those that withdrew) viewed microsystems as a commercial enterprise that re-packaged old ideas and was overly complex. Practices with a more positive outlook carried out further projects but adapted the process, using the tools they found helpful and discarding others. The need to keep the approach visible, promote ownership and maintain enthusiasm was highlighted:

*You’ve got graphs on the wall, of how much we were achieving, so every time somebody came in to make a cup of tea they felt like they wanted to contribute to making that a success, but now we’ve got nothing visual, and people don’t actually know what’s going on… we’ve lost the momentum* (P1, PM, early adopter)

Some practices (and coaches) felt that they needed a refresher, perhaps 6 to 12 months later, to help embed into practice, review progress, address problems and upskill.

## Discussion

This was a small evaluation, and the main study limitation was recruiting sufficient GPs particularly those who had not participated or withdrew. Although anticipated, this resulted in significant delays with data collection. It was difficult to engage more than one person per practice which limited the range of perspectives represented in the data and may have resulted in selection bias. We used Rogers (2010) and NPT (May *et al*., 2009) as a framework to explore implementation but it was difficult to elucidate which contextual trigger led to exactly what outcome and why, more the remit of realist methodology (Pawson, 2006). Additionally, the data only represent a ‘snapshot’ in time when systemic change in primary care is rapid and ongoing.

The findings explored issues around implementation, embedding into practice and sustainability of the CMS approach. Early adopters were able to identify benefits and perceived the approach as an effective method of addressing discreet and process-driven issues within GP. The late majority had mixed views, but small projects with a stable core team and clear parameters were perceived as more successful than bigger ones with a larger team, wider remit and crossing microsystems. This is clearly intuitive given the basic concept of CMS, but some participants had strong rationale for working across boundaries, or in the realm of mesosystems, but encountered cross-organisational barriers that made it difficult to sustain projects. Although CMS are intended to work across all system levels (Nelson *et al*., 2008), other studies have identified difficulties with cross-boundary working (Williams *et al*., 2009; Dunham *et al*., 2018; Gerrish *et al*., 2018), not least the impact of organisational turbulence (Gerrish *et al*., 2018). Dunham *et al*. (2018) found that even strong mesosystem support was insufficient to guarantee change unless coupled with strong structures and processes in the CMS, which they attributed to high-quality training.

Although the CCG offered training and reimbursement, this was insufficient an incentive for those that withdrew. Critically, the approach did not appear to self-sustain. Few practices had embedded the use of the CMS methodology and related this to time, staffing and competing priorities. The drive and enthusiasm of an external coach were often cited as key to maintaining momentum and once the coach left, and reimbursement ceased, commitment waned. Although the CMS toolkit was useful, in that it facilitated a methodical process, in subsequent projects participants tended to ‘streamline’ it, inadvertently compromising fidelity (Chaudoir *et al*., 2013). Drawing on NPT (May *et al*., 2009), Figure 2 provides a visual overview of key issues.

**Figure 2.**
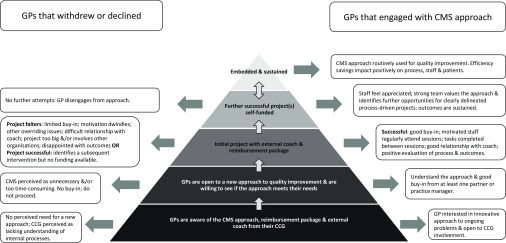
Implementing, embedding and sustaining the CMS approach within general practice.

Our findings identified how the interplay between individual attributes, relationships within the practice, with the coach and CCG, and with other organisations all affected successful implementation. This resonates with Damschorder *et al*.’s (2009) CFIR framework and factors within the inner and outer setting (or micro- and meso/macro-level). For example, individuals with a positive attitude to change working in a team with good morale (inner setting) and a positive relationship with their coach and CCG (outer setting) identified meaningful outcomes. CMS appeared able to address factors at one level, for example individual buy-in, but appeared unable to break through multiple barriers, such as team discord and cross-organisational projects. Drawing on NPT (May *et al*., 2009), individual and/or team resistance to buy-in appeared pivotal, based on concerns that it was a ‘top-down’ approach imposed on them, although CMS actually employs a ‘bottom-up’ approach to enable staff to identify their own QI priorities (Gerrish*et al*., 2018).

Given that the key benefits (relationship with the CCG, relationship within the practice and time to reflect) are unlikely to be unique attributes of the CMS approach, as opposed to other QI programmes, this raises doubt about the added value of the CMS approach per se. In the context of major service reconfiguration (NHS England, 2019b), working across boundaries is essential but few participants appeared to have considered CMS as a key contender for addressing this challenge. Similarly, there was no evidence that participants perceived CMS as a tool to equip them to meet the complex macro-level challenges that are facing GPs including: an ageing population with multiple morbidities; workforce recruitment and retention issues; policy focused on primary prevention and integrated working; and financial constraints (NHS England, 2019b).

Recent NHS England guidelines for QI in GP (NHS England, 2019c) recommend that GPs set their own areas for improvement yet relate specifically to (imposed) QOF domains. Our evaluation has demonstrated that for the CMS approach to succeed, it has to have buy-in from all parties and this is least likely when priorities are imposed. Moreover, the guidelines refer to a QI guide (Royal College of General Practitioners, 2015) comparable to the CMS toolkit but similarly not designed to meet the challenge of working in PCNs. Finally, the PDSA cycle, a key component of many QI approaches including microsystems, is often poorly understood and implemented (Taylor *et al*., 2014), potentially compromising fidelity.

In conclusion, our findings suggest that CMS relies on: establishing a contract between coach and GP to promote buy-in; a flexible approach; identifying a champion at the outset; the use of outcome measures that demonstrate not only the impact on process and systems but also the wider benefits for staff and patients; and follow-up after completion of a project to boost motivation, review progress and refresh skills. Further research at this level should explore how to build sustainability so that changes become routinised, how to embed the approach into practice and how to evidence less tangible outcomes, such as team cohesiveness. Given the anticipated size of PCNs and the number of stakeholders involved (NHS England, 2019b), it is unlikely that CMS, or similar approaches, are suited to the challenge of major organisational mergers. The study did not intend to compare CMS to other non-proprietary QI approaches but the findings suggest that future research should focus on a comparison of approaches to identify which, if any, are suited to cross-organisational working and what, if any, added value the CMS approach provides. However, this does not negate the benefits of using CMS for small process-driven projects within an organisation where it provides a systematic approach to streamlining GP processes, essential in the current climate.

## Supplementary material

For supplementary material accompanying this paper visit http://dx.doi.org/10.1017/S1463423620000158. click here to view supplementary material
