# Supplementary material for: An evaluation of the clinical microsystems approach in general practice quality improvement
Source: Prim Health Care Res Dev. 2020 Jun 23;21:e21. doi: 10.1017/S1463423620000158 (PMC7327435; doi:10.1017/S1463423620000158)
Supplement: Supplementary file 1 [file S1463423620000158sup.zip › S1463423620000158sup003.docx]

**Supplementary information 3: Topic guide for coaches**

| **NPT area** | **Question & prompts**  Abbreviation: clinical microsystems programme (CMP) |
| --- | --- |
|  | **To start off, could you tell me a little about yourself?**   1. Your role and how long you have been at the CCG 2. Your knowledge and/or experience of CMPs prior to training as a coach? 3. How long have you been coaching in this capacity? How many projects you have facilitated? Can you describe them to me? |
| Sense making/ coherence | **When you started coaching, what were your expectations for the CMP?**   1. Were there any particular influences on the decision to fund the programme? 2. What were you hoping the programme would bring to general practices? 3. Why did you feel there was a need for the programme? 4. How did you select which GP practices to work with? 5. How were you prepared for the role of coach? |
| Participation/ engagement | **Was there “buy-in” to the programme within the CCG?**   1. As a membership organisation were all CCG members happy to be involved in the CMP? Did this change over time? Can you give me an example? 2. What is involved in funding the CMP? What is actually being funded? 3. Were there any concerns about the enhanced service payment? 4. Do you think all commissioners understood the potential value of the programme? 5. Was there any conflict between your role as commissioner and being a coach?   **Thinking about the projects you’ve facilitated, was there “buy-in” from practice staff?**   1. Were all staff in the practice happy to be involved in the CMP? Did this change over time? Can you give me an example? 2. Did you find that someone in the practice needed to champion the programme? Can you give me an example? 3. How important is the enhanced service payment for buy-in? Do you think it needs to be continued to sustain buy-in? 4. Were you involved with any practices that resisted involvement or decided to withdraw? *Ask to expand on rationale* |
| Action/ doing | **Now, on to what the programme actually involved for you and practices**   1. What does the programme actually require the practice to do?   How much time does this take?  What kind of activities did they have to undertake?  What do you think are the benefit of the 5Ps mapping process? And other tools?  Were there any specific enablers or barriers?   1. How would you describe your approach to coaching? [*philosophy*]   What does it actually involve for you? [*logistics*]   1. How would you describe the relationship between the coaches and practices [*global*]? Can you give me an example from your own coaching?   How often do you see them when working on a project?  How do you maintain contact?  Were there any specific enablers or barriers? |
| Appraisal | **Finally, what value do you think the programme provided**   1. How would you describe the changes the programme bought for practices? Can you give me some examples? 2. How do you think the programme equips practices for future challenges? 3. Who has benefited most from the programme and how? 4. Would you recommend the programme to other CCGs? If yes, what are the “key ingredients” for the programme to run effectively? Can you elaborate? 5. Do you think that the CCG will continue to support the CMP? 6. As a whole, how could the CMP be improved? |
