# Supplementary material for: An evaluation of the clinical microsystems approach in general practice quality improvement
Source: Prim Health Care Res Dev. 2020 Jun 23;21:e21. doi: 10.1017/S1463423620000158 (PMC7327435; doi:10.1017/S1463423620000158)
Supplement: Supplementary file 1 [file S1463423620000158sup.zip › S1463423620000158sup002.docx]

## Supplementary information 1: Summary of studies appertaining to clinical microsystems

| Author | Setting | Aims | Methodology | Sample size | Key findings |
| --- | --- | --- | --- | --- | --- |
| Literature relating to general practice and/or UK healthcare context (most recent first) | | | | | |
| (Dunham et al. 2018) | General practices, metropolitan and rural, **Australia** | To identify the success attributions of high performing Australian general practices. The attributes were compared to the framework of success characteristics in microsystems. | Qualitative/ interpretive. Semi-structured interviews and content analysis. | Twenty-two general practices identified as high performing (using 10 success criteria). The 52 participants were 19 GPs, 18 practice managers and 15 practice nurses. | Participants most frequently attributed success to inclusive leadership, interdependence of the team, patient focus and staff focus. Honesty and trust displayed by team members helped to create ‘cultures of learning and improvement’ whereby the team could collectively improve things. Barriers were couched in terms of deficits in, or limitations of the success characteristics. Structures and processes at practice level govern delivery of care. |
| (Baird et al. 2018)  *Same study as Risi (2015), below* | General Practice, **UK/ international** | To explore different delivery models in general practice that could address current challenges in UK general practices. | Report based on literature review and interviews with a variety of stakeholders. Case studies include Tower Hamlets’ pilot of micro-teams within general practice. | Not specified. | The overall report developed a set of core attributes of general practice. The evaluation of Tower Hamlets reported mixed progress with only one micro-team model achieving a significant degree of success. Early findings found that the approach could improve safety, reduce GP workload by avoiding duplication of effort and improve co-ordination.  Lessons included: involving patients from the start; engaging the whole practice team (buy-in from all GPs was vital and staff turnover detrimental); infrastructure to support teams including how to manage work that fell between teams; investment in staff training; external input to support change management. |
| (Risi et al. 2015) | General Practice, Tower Hamlets, **UK** | Summary of a microsystem approach in general practice to address a) delays in cancer diagnoses and b) lack of continuity of care in the year preceding death in hospital. | Opinion piece | Five practices were initially involved. Staff were to be interviewed to monitor progress but no details are provided. | Anecdotal evidence suggests that micro-teams can bring back the best aspects of small practice working but under the protective umbrella of being part of a larger team (a newly established GP federation of all practices in Tower Hamlets). Benefits included opportunity for peer review of complex cases, improved safety through a second opinion and emotional support for staff. |
| (Janamian et al. 2014) | The Sunshine Coast Division of General Practice (SCDGP), Queensland, **Australia** | Evaluation of an Improved Diabetes Management (IDM) programme. Aims included determining the role of the clinical microsystem approach in triggering the successful adoption of the programme and identifying barriers to implementation. | Qualitative evaluation with purposive sample. | Five key informants and 5 GPs. | The identification of champions of change, the celebration of positive achievements and the use ‘real data’ to demonstrate improved health outcomes for patients from the practice were instrumental in motivating participating GPs to both implement and sustain changes in their diabetes care delivery. The microsystems approach offered a means of integrating structure, process and outcomes of a care framework for reviewing improvements in the delivery of care. |
| (Michael et al. 2013) | Adult Primary Care Unit (ambulatory healthcare in a county health department), Florida, **USA** | Goals included: to identify factors contributing to long wait times; to minimise wait times; and to evaluate the impact of the microsystems approach on patient wait times, patient satisfaction with wait times and with overall care. | Quality improvement project, 8 phases. Included tracking pre- and post-test to compare wait times for waiting room wait times and examination room wait times. | Overall 1365 patients’ wait times were tracked. First patient survey: 262 returned; second survey 285 returned (response rate of 42% and 47% respectively). | Mean waiting room wait time for patients seen during the post-implementation period were slightly reduced but while statistically significant, targeted wait time goals were not met. The patient satisfaction scores were significant in the waiting room wait time category (*p* = .029) but not for the exam room wait time. The results support the use of the microsystems approach (including Plan-Do-Act-Study, PDAS) as viable options for conducting quality improvement. |
| (Gobel et al. 2012)  *Part of a larger project which studied the hospital-community interface in 6 countries.* | Hospital to community interface, Netherlands. | To apply a microsystem lens to gain insights into gaps in the handover process from acute care to the general practitioner, and to develop recommendations for improving handovers between primary and secondary care. | A qualitative thematic analysis using a clinical microsystems lens. | 28 semi-structured key stakeholder interviews (7 patients/21 professionals) that constituted seven complete microsystems (a patient, a hospital physician, a hospital nurse and GP a). | Five themes emerged related handovers: (1) lack of adequate information; (2) healthcare professionals’ availability and opportunity for personal contact; (3) feedback, teaching and protocols related to handovers; (4) IT facilitated communication solutions; and (5) the role and responsibility of the patient.  Comments on the lack of standardisation, coordination and training for handover were consistent. Healthcare professionals seemed to have difficulty contacting and communicating with each other, and worked in isolation. A possible explanation may be an inability to grasp the interdependencies of the system. Professionals can be proficient in their own clinical domain but may not appreciate their impact on the larger system and its impact on patient outcomes. The study offered an innovative approach to assessing and addressing the gaps between current handover practices from the hospital to community by viewing this interface as a virtual microsystem. |
| (Williams et al. 2009)  *Same study as Williams et al (2007), below* | England, six National Health Service (NHS) sites. | To evaluate the claims made for the clinical microsystems approach of healthcare improvement within an NHS context. | Realist evaluation, six case study sites, mixed methods. | Does not specify numbers of interviews or outcome data. | The findings resonated with many of the claims for clinical microsystems, particularly that democratic, consensual approaches to change and improvement can be better received than externally derived initiatives with imposed targets. The microsystem approach emphasises identifying and nurturing strengths, of both teams and individuals, and this reinforced these positive aspects. The case study sites demonstrated higher staff morale, empowerment, commitment and clarity of purpose. However, future microsystem programmes need to address components of patient involvement and process/outcome monitoring. |
| (Williams, Dickinson and Robinson 2007) | England, NHS, primary and secondary care. | Two main aims were: to gain feedback on the developing role of clinical microsystems in the strategy for building local improvement capability; and to understand the value of microsystems in providing spread, sustainability and service transformation. | Realist evaluation. Six case study sites across England: genito-urinary clinic, occupational therapy service, community mental health team, cardiac rehabilitation, surgical & medical wards and smoking cessation service. | Interviews and discussions: does not specify. Outcome measures collected per site: not stated, other than that they found a ‘paucity’ (p14) of routine data collection. | Perceived benefits included improved communication within the microsystem; better team morale; greater awareness of the service’s function and individual roles in delivering these; a shift in culture towards a more active approach to individual and collective improvement; and a greater capacity to manage externally imposed change. Some respondents did not buy-in to the process: the reasons were unclear but included scepticism and disliking the terminology. The overall focus was on staff (‘people’) and process which was reflected in the relative absence of outcomes of patients and lack of measurable impact on quality, safety, productivity or efficiency. |
| (Nemeth et al. 2008) | USA, Primary care practices | To explore the process of change used to implement clinical guidelines for primary and secondary prevention of cardiovascular disease in primary care practices that used a common electronic medical record (EMR). | Qualitative, interviews. Part of a larger process evaluation. | Purposive sampling in eight primary care practices within the larger clinical trial. Interviews with 28 staff and clinicians. | The larger study used multiple conceptual frameworks primarily that of microsystems, to inform the intervention (implementing guidelines for cardiovascular disease). Microsystems provided a mechanism to drill deeper into the meaning of the process of change and this led to a new framework for implementing change that elucidated seven concepts:  1. Vision with clear goals  2. Team involvement  3. Enhance communication systems  4. Develop staff knowledge  5. Take small steps  6. Assimilate EMR into clinical practice  7. Feedback within a culture of improvement  The qualitative findings were helpful in explaining how the results of performance improvements were accomplished. |
| (Rhydderch et al. 2005) | NA | To inform the debate on the use of organisational assessments in general practice. | Systematic review of international-peer-reviewed literature. | Thirteen papers describing five organisational assessment instruments. Included Mohr & Batalden (2002), see below. | Useful comparison of externally led quality assurance versus internally led quality improvement which is regarded as a continuum relative to criteria including whose agenda, the emphasis and the mechanism of assessment.  Microsystems combine complexity and systems theories by combining the principles of measurement and feedback to provide data to stimulate team-based solutions. The aim is to move forward incrementally, continually improving and therefore raising minimum and maximum standards (Mohr & Batalden 2002). However, the approach lacks data on reliability and validity. |
| Studies related to microsystems but either non-UK or not general practice | | | | | |
| (Pandhi et al. 2018) | A large academic health care system, Wisconsin, USA | To assess the impact of clinical microsystems approach on 1) team members perceptions of the impact at 6 and 12 months and 2) what challenges occurred during implementation and how they were addressed. | Longitudinal survey augmented by interviews and focus groups. | 58 primary care teams; 204/257 individuals completed the baseline survey across all the teams (range 49-92%). Completion rates at 6 and 12 months were 45-77% and 52-81% respectively. | Survey results indicated improved perceptions of organizational support; team effectiveness and cohesion; quality improvement skills; and team communication. Thematic challenges from the qualitative data included: lack of time; need for technical support; tensions between team and clinic level change; a part-time workforce; and team instability.  The findings suggested that a microsystems approach is valuable for building team relationships and quality improvement skills but is challenged in a large, diverse academic primary care context. Also suggests that primary care transformation requires purposeful changes implemented across the micro to macro-level including but not only focused on quality improvement training for microsystem teams. |
| (Gerrish, Keen and Palfreyman 2018) | Sheffield, UK, three discrete community services | To identify learning from a clinical microsystems quality improvement initiative  to develop a more integrated service across a falls care pathway spanning community and  hospital services. | Quality improvement programme using microsystems approach across the Falls Care Pathway. |  | Divided into three phases: developing a climate for change; implementation; and achieving change. The initiative was successful in delivering change in relation to key aspects of the pathway, engaging frontline staff and decision makers from different services. Viewing the pathway as a series of interrelated microsystems enabled stakeholders to understand the complex nature of the pathway and to target key areas for change. Particular challenges encountered arose from organisational reconfiguration and cross-boundary working. Recognition of the pathway operating at meso- and macrosystem levels fostered wider stakeholder engagement with the potential of improving integration of care. |
| (Likosky 2014) | Australia, acute cardiac care | To demonstrate how clinical microsystems can be used to support improvement in the delivery of care, including methods for engaging teams in clinical redesign. | Opinion and case example (2002) which evaluated | The case example is described elsewhere: the impact of operative practices on mechanisms of brain injury after cardiac surgery. | Hospitals and healthcare organizations are made up of hundreds of microsystems. The challenge is to identify the microsystem(s) in which we work every day and strive to maximise their function. It is also important to consider how a given microsystem relates to other microsystems within an organization and its overall strategic aims. This approach is an important mechanism to improve efficiency and reliability. |
| (O'Dwyer 2014) | Ireland, emergency department | To describe the implementation of a microsystems approach into an emergency department and the resultant impact on patient care. | MSc thesis using models of change | Several different strands including on-line survey with staff; SWOT analysis; and patient data. | Successfully implemented with positive outcomes for patients and staff: the microsystems improved patient care and allowed ownership of quality improvement initiatives by staff in the department. |
| (Gill and Gray 2006) | Humber Mental Health Teaching NHS Trust | Description of a service improvement programme using microsystems with three community mental health teams. | Report (unpublished) | Not stated | The authors argue that microsystems were an effective method for engaging front line teams in a mental health setting. The mesosystem acts as a mediator between microsystems and the strategic imperatives of the wider NHS; gaining an understanding of both can lead to more effective working. Mesosystems are described as ‘a semi-permeable membrane between the microsystems and the macrosystem’. Mesosystem regarded as the management layer (e.g. heads of services). |
| Studies appertaining to microsystems theory and/or education | | | | | |
| (Gerard, Grossman and Godfrey 2012) | USA, clinical nurse leader (CNL) education. | To share aspects of course development for the role of CNL and active learning experiences used for CNL development. | Discussion piece | NA | Key components of the course are described in detail including the ‘5P’s clinical microsystems assessment. Students carried out the 5Ps and this led to a new understanding of a familiar clinical area. The principles of collaboration and partnership, integral to the microsystem approach, fitted well with the CNL role as leader of the interdisciplinary team and agent of change. |
| (Nelson et al. 2008) | USA healthcare system. | **Part 1** in a four-part series building on the original nine-part series on clinical microsystems in health care. Summarises lessons learned and addresses second-generation microsystem development. | ‘Lessons from the field’ | NA | For a health system to work everyone in the system needs to help achieve three fundamental needs: better patient outcomes, better system performance, and better professional development.  A mesosystem refers to a collection of interrelated microsystems that provide care to a shared population of patients. One role of the mesosystem is to guide dialogue between related microsystems to achieve patient outcomes and to feed information in both directions.  Barriers include: lack of data e.g. benchmarking information) which obscures performance gaps; individual attitudes (e.g. eschewing personal responsibility); and resources to engage and assist frontline staff. |
| (Wasson et al. 2008) | As above | **Part 2** of the above series focuses on patient needs, process improvement and routinely measure patterns of performance, and feedback data. | ‘Lessons from the field’ and case examples | Case examples from primary care in ambulatory community settings | Exemplar microsystem will a) have as its primary purpose a focus on the patient b) commitment to process improvement including study, measurement and improvement of care and c) routinely measure its patterns of performance, or feedback data. Patients should be able to report that they receive “exactly the care they want and need exactly when and how they want and need it.”  Barriers to learning from micro practices include a) failure to promote leadership, culture, organisational support, staff focus, and interdependence of the team b) failure to develop an adaptable team of the right size and c) resistance to change or inertia; regulatory approaches based on payment by result may impede development of the microsystem. |
| (Godfrey et al. 2008) | Inpatient care, USA. | **Part 3** describes the transformation of two hospitals using micro-, meso- and macro-system strategies. | Commentary | Case examples from one large urban academic children’s medical centre and one rural community hospital. | The development of high-functioning clinical microsystems emerged over the same time as other important changes, including the development of improvement infrastructure, the availability of outcome and process data at the microsystem level, and transparency of improvement prioritisation at all levels of the organisation. It was not possible to single out any one individual change that resulted in the transformation.  The dialogue to negotiate improvement at all levels is the “back and forth” between macro/ meso/ microsystems to find the right balance to meet the organisation goals while identifying the capacity and ability of the micro- and mesosystems to lead and spread improvement. Mesosystem leaders learnt the crucial importance of aligning improvement goals with operational expectations. Linked closely to sustaining the gains is how measurement is built into the micro-, meso-, and macrosystems. |
| (McKinley et al. 2008) | As above | **Part 4** describes how adaptation of the microsystem framework led to a novel model of care delivery for patients requiring elective coronary artery bypass surgery. | Mix of case description and output data over 3 years. | Case example: acute cardiac surgery. | Developed a framework which specified three key areas of focus for organisations to achieve system-level results: system-level goals, local management and supervision, and workforce development. Professionals from many microsystems and supporting hospital services continuously revolve around the patient. Professionals from these microsystems and services oscillate within a certain proximity of the patient during a given hospital stay. At times, the professionals and services are very close to or occur within the microsystem where the patient is receiving care, and at times the work done for the patient occurs without direct interaction with the patient. The sum of the interactions between the microsystems, hospital services, and professionals revolving around the patient is the newly formed mesosystem. |
| (Foster et al. 2007) | NA | Comparison of Baldrige criteria for organisational quality assessment and improvement with microsystems characteristics. | Discussion paper based on interviews with members of 20 high-performing microsystems. | Described in prior paper (Nelson, 2002, Part 1,) | Both Baldrige criteria and microsystem success characteristics cover a wide range of areas crucial to high performance. Those identified from a Baldrige standpoint were organisational leadership, work systems and service processes. Microsystem characteristics for success are leadership, performance results, process improvement, and information/ information technology. |
| (Mohr and Batalden 2002) | North America | Description of microsystems, their characteristics and operational definitions, and an assessment tool. | Discussion paper/ description of tool based on previous qualitative research (interviews with representatives from 43 microsystems across North America). | NA. The interviews are described elsewhere. | A clinical microsystem is a small organised group of clinicians and staff working together with a shared clinical purpose to provide care for a defined set of patients. Use of information technology facilitates collecting, assessing, and sharing of information. Microsystems are usually part of a larger organisation and are embedded in a legal, financial, social, and regulatory environment. Eight characteristics were identified:   - Integration of information - Measurement of process and outcomes - Interdependence of the care team - Supportiveness of the larger system - Constancy of purpose - Connection to the community - Investment in improvement - Alignment of role and training |
| (Nelson et al. 2002; Nelson et al. 2003; Wasson et al. 2003; Mohr et al. 2003; Kosnik and Espinosa 2003; Huber et al. 2003; Batalden, Nelson, Edwards et al. 2003; Batalden, Nelson, Mohr et al. 2003) | USA. The original series of 9 articles by the key proponents of microsystems:  1: Learning from high-performing front-line clinical units  2: Creating a rich information environment  3: Planning patient-centered services  4: Planning patient centred care  5: How leaders are leading  6: Designing patient safety into the MS  7: The Microsystem as a platform for merging strategic planning & operations  8: Developing people & improving work life: what frontline staff told us  9: Developing small clinical units to attain peak performance (above) | | | | The authors identified and sampled 20 of the best-value small clinical units in North America, 2000-02. The series are based on case studies to illustrate the microsystem approach. Sites were screened and selected using a self-administered survey, telephone interview, and two-day site visits for in-depth interviews and observations. There were 4 primary care practices, 5 medical specialty practices, 4 inpatient units, 4 home health care units and 3 nursing home and hospice facilities. |
