# Supplementary material for: An evaluation of the clinical microsystems approach in general practice quality improvement
Source: Prim Health Care Res Dev. 2020 Jun 23;21:e21. doi: 10.1017/S1463423620000158 (PMC7327435; doi:10.1017/S1463423620000158)
Supplement: Supplementary file 1 [file S1463423620000158sup.zip › S1463423620000158sup001.docx]

**Supplementary information 2: Topic guide for GPs that withdrew or declined**

1. **Practices that withdrew from a CMS programme**

| NPT area | Question & prompts |
| --- | --- |
|  | **To start off, could you tell me a little about yourself?**   1. Your role and how long you have been at the practice 2. Your knowledge and/or experience of clinical microsystems prior to the programme 3. Your involvement in the clinical microsystems programme and what activities you carried out up to the decision to withdraw (probe re 5Ps etc) [***Action/doing***] |
| Sense making/ coherence | **What were your expectations for the clinical microsystems programme?**   1. What were you hoping the programme would bring to the practice? 2. Why did you feel there was a need for the programme? 3. Did you feel well-prepared about what it would involve for the practice? 4. In what way were your expectations not met? |
| Participation/ buy-in | **What issues led to your withdrawing?**   1. Were there any staff who voiced concerns about the programme? What do you think their reasons were?   **Did you discuss your concerns with the coach?**   1. How were they addressed? 2. What was the relationship between your practice and the coach? 3. Could the coach have done anything differently? 4. **Did the enhanced service payment influence any thinking** about joining or withdrawing from the programme? |
| Appraisal | **What could be changed in the programme to make it useful for your practice?**   1. How could the programme be improved? 2. Would you recommend the programme to others? |

1. **Practices that did not undertake the programme**

| NPT area | Question & prompts |
| --- | --- |
|  | **To start off, could you tell me a little about yourself?**   1. Your role and how long you have been at the practice 2. Your knowledge and/or experience of clinical microsystems prior to the programme |
| Sense making/ coherence | **What were your expectations for the clinical microsystems programme?**   1. What were the reasons the practice decided not to participate in the programme? 2. What were the needs of your practice that you felt the programme could not address? 3. Were you happy with the information you received about the programme prior to making your decision? 4. Were there any particular staff who voiced concerns about the programme? What do you think their reasons were? 5. What would have encouraged you to participate? |
| Participation/ engagement | 1. Did the enhanced service payment influence your decision in any way? |
| Appraisal | 1. What could be altered that might change your view of the programme? |
